# Non-Invasive Diagnostic Test for Advanced Fibrosis in Adolescents With Non-Alcoholic Fatty Liver Disease

**DOI:** 10.3389/fped.2022.885576

**Published:** 2022-04-26

**Authors:** Antonella Mosca, Luca Della Volpe, Anna Alisi, Silvio Veraldi, Paola Francalanci, Giuseppe Maggiore

**Affiliations:** ^1^Hepatogastroenterology, Nutrition, Digestive Endoscopy and Liver Transplant Unit, Bambino Gesù Children's Hospital, IRCCS, Rome, Italy; ^2^Research Unit of Molecular Genetics of Complex Phenotypes, Bambino Gesù Children's Hospital, IRCCS, Rome, Italy; ^3^Pathology Unit, Department of Diagnostic and Laboratory Medicine, IRCCS Bambino Gesù Children's Hospital, Rome, Italy

**Keywords:** NAFLD, children, NASH, fibrosis, fibrosis score

## Abstract

**Introduction:**

Non-alcoholic fatty liver disease (NAFLD) is a multifaceted disease that includes a wide spectrum of liver damage. The presence and the degree of fibrosis are considered important factors for the prognosis of NAFLD and in predicting the risk of developing cirrhosis. Our aim was to evaluate the usefulness of four fibrosis scores (aspartate aminotransferase/Platelet Index [APRI], FIB-4, NAFLD Fibrosis Score [NFS], and Hepamet) in predicting different degrees of fibrosis among children with biopsy-proven NAFLD.

**Methods:**

About 286 adolescents [mean age 14.3 years ± 2.5; 154 (53.6%) males], referred between January 2014 and December 2019, with biopsy-proven NAFLD were enrolled.

**Results:**

About 173 (60.4%) patients presented fibrosis at histological analysis. In particular: 140 (49.3%) patients had *F* = 1, 31 (10.8%), had *F* = 2 and 2 (0.66%) had *F* = 3. APRI (AUROC 0.619, 95% CI 0.556–0.679) and Hepamet (AUROC 0.778, 95% CI 0.722–0.828) scores had significant (*p* < 0.001) accuracy to distinguish subjects with fibrosis; while NFS and FIB-4 had not. APRI had a positive predictive value (PPV) of 62.77% (95% CI 57.96–67.35) and an negative predictive value (NPV) of 52.01% (95% CI 46.54–57.43); Hepamet a PPV of 63.24% (95% CI 59.95–66.41) and an NPV of 61.29% (52.9–69.01).

**Conclusions:**

Our study showed that Hepamet and APRI perform better than NFS and FIB-4 for identifying fibrosis in patients with NAFLD, but do not have PPVs so high to be considered diagnostic. Therefore, they cannot be employed, in children, for a certain diagnosis of fibrosis or its progression and cannot replace liver biopsy as the gold diagnostic standard. It is, therefore, necessary to continue to research and develop new markers of exclusive fibrosis.

## Introduction

Non-alcoholic fatty liver disease (NAFLD) is a multifaceted disease that includes a wide spectrum of liver damage, ranging from the accumulation of fat in more than 5% of hepatocytes (NAFLD) to non-alcoholic steatohepatitis (NASH), characterized by tissue necroinflammation and possible fibrosis at different stages associated with steatosis (1). NAFLD, as a feature of metabolic syndrome, is a cause of increasing concern in pediatrics, and it is estimated to affect 5–10% of children and adolescents in westernized countries (2). Several studies suggest that NASH may progress to cirrhosis and end-stage liver disease that requires liver transplantation. Over the past 20 years, researchers have investigated how NAFLD and NASH follow an aggressive course in children, with data showing that many children progress to fibrosis and advanced cirrhosis as early as childhood or early adulthood (3–5). A recent study conducted on 867 adolescents with NAFLD found that 4.4% of children had significant fibrosis (>F2) at transient elastography evaluation (3). The presence and stage of fibrosis are considered important factors in the prognosis of NAFLD and in predicting the risk of developing cirrhosis (5, 6).

To date, the gold standard of diagnosis remains percutaneous liver biopsy (7). However, a biopsy is not only an expensive procedure, but it is also invasive with a high risk of complications and sampling errors. Therefore, it became necessary to investigate new non-invasive tests for early diagnosis of fibrosis in pediatric NAFLD as they may play an important role in preventing the development of further complications (6, 7). Elastography techniques are a range of methods that non-invasively assess liver stiffness through the measurement of the velocity of the propagation of a shear wave generated by a probe. These techniques have proved to be reliable and reproducible methods to estimate fibrosis in children and adolescents, even if may be limited by the presence of obesity, severe congestion, and inflammation (8).

Recently, there has been an increased search for non-invasive scores to diagnose the stage of NAFLD fibrosis (9–12). Many of the fibrosis scores have been developed and validated in large studies on adult with NAFLD, and are part of routine clinical practice, including the AST/Platelet Index (APRI), FIB-4 score, and NAFLD Fibrosis Score (NFS) (11, 13).

A new non-invasive fibrosis-Hepamet score has been developed and validated in adults with NAFLD and incorporates simple anthropometric and laboratory parameters to predict fibrosis and advanced cirrhosis. Hepamet would have greater accuracy and fewer indeterminate results than FIB-4 and NFS, and most importantly the test is not affected by age or BMI (13, 14). However, none of these scores have been validated in children with NAFLD (15). Our aim was to evaluate the usefulness of these fibrosis scores in predicting different stages of fibrosis among children with biopsy-proven NAFLD.

## Materials and Methods

We enrolled 286 adolescents [mean age 14.3 years ± 2.5; 154 (53.6%) males], with an ultrasound diagnosis of severe hepatic steatosis and/or for insistently (≥6 months) elevated serum aminotransferase levels, who were consecutively referred to the liver unit of “Bambino Gesù” Children Hospital (Rome) between January 2014 and December 2019, and those who agreed to undergo liver biopsy for diagnosing the severity of NAFLD in accordance with ESPGHAN guidelines (7). Most of these adolescents were overweight or obese. All adolescents were tested to exclude secondary causes of hepatic steatosis (e.g., Wilson's disease, α-1-antitrypsin deficiency, viral hepatitis, autoimmune hepatitis, endocrinological, genetic, and metabolic diseases, celiac disease, alcohol, and drug consumption) (7, 16). All were Caucasians of Italian origin. The study was carried out according to the rules of the Helsinki Declaration.

Body mass index (BMI) and waist circumference (WC) were calculated as previously described (17). Blood pressure was measured in the right arm pressure using a standard sphygmomanometer; the mean of three blood pressure values was reported. Elevated blood pressure has been defined by systolic or diastolic blood >95th percentile for age, height, and sex. Venous blood samples were collected after a fast of at least 8 h. Serum liver enzymes (aspartate aminotransferase [AST], alanine aminotransferase [ALT], and gamma-glutamyltransferase), lipids [total cholesterol, high-density lipoprotein cholesterol, low-density lipoprotein -cholesterol and triglycerides], platelet counts, albumin, fasting glucose and insulin levels were measured in all patients using the standard laboratory procedures at the Central Laboratory of the “Bambino Gesù” Children Hospital. Homeostasis evaluation of the model score was used for the estimation of insulin-resistance (HOMA-IR), a value >2.5 was considered as an index of insulin resistance (18).

Liver biopsies were performed using an automatic 18 fr caliber biopsy needle, general anesthesia, and guided ultrasound. The characteristic histological features of NAFLD were steatosis, portal and lobular inflammation, balloon hepatocyte, and fibrosis. A single experienced liver pathologist evaluated all liver biopsies. NASH was characterized by the scoring system developed by the National Institutes of Health NASH Clinical Research Network (19). Hepatic steatosis was classified on four scales: 0 = steatosis involving less than 5% of hepatocytes, 1 = steatosis involving up to 33% of hepatocytes, 2 = engaging steatosis = 33–66% of hepatocytes, and 3 = steatosis of 66% of hepatocytes. Lobular inflammation was classified on four scale points: 0 = n foci, 1 = less than two foci per 200× field, 2 = two to four foci per field 200×, and 3 = more than four foci per field 200×. Portal inflammation was classified on four scale points: 0 = none, 1 = mild, 2 = moderate, and 3 = severe. Hepatocyte balloon was classified on three scale points: 0 = no balloon cells, 1 = few balloon cells, and 2 = many/prominent balloon cells. The stage of hepatic fibrosis was quantified using a five-point scale: 0 = fibrosis,1 = perisinusoidal or periportal fibrosis [(1a) mild, zone 3, perisinusoidal;(1b) moderate, zone 3, perisinusoidal; and (1c) portal/periportal], 2 = portal perisinusoidal/periportal fibrosis, 3 = fibrous bridge, and 4 = cirrhosis (19, 20).

### Calculation of Non-Invasive Fibrosis Scores

We calculated four non-invasive fibrosis scores: APRI, Hepamet, FIB-4, and NFS. The AST to Platelet Ratio Index score is equal ALT(U/L) /AST(U/L)^*^100/platelet count(10^9^) NFS and FIB-4 scores were calculated using the original formulas. NFS: −1.675 + 0.037 × age (years) + 0.094 × BMI (kg/m2) + 1.13 × T2DM/IGF (yes = 1, no = 0) + 0.99 × AST/ALT ratio−0.013 × platelet count (x109/L)−0.66 × albumin (g/dl). FIB-4 score: age (years) × AST (U/L) / platelets (109/L) × √ALT (U/L). The Hepamet score was calculated using a free online application (https://www.hepamet-fibrosis-score.eu/) (13, 14, 21). The pediatric NAFLD fibrosis index (PNFI) was calculated used the original formulas showed in Nobili et al. (15) (http://www.giorgiobedogni.it/faq/pfaq.html).

### Statistical Analysis

The data are expressed as mean and SD (standard deviation) or as medians and interquartile intervals (IQRs) or frequencies. Differences in clinical variables were tested by the exact Fischer test for categorical variables, by unidirectional ANOVA for normally distributed continuous variables, and by the Kruskal–Wallis test for non-normally distributed continuous variables. Spearman correlation coefficients were calculated to examine the invariable linear association.

A *p* < 0.05 was considered statistically significant.

The overall diagnostic accuracy was assessed by determining the area under the ROC curve (AUROC). Diagnostic performance was determined by sensitivity, specificity, positive predictive value (PPV), and negative predictive value (NPV). The two-tailed *p* < 0.05 were considered statistically significant. The ROC curves were compared according to the Hanley and McNeil method and the performance of the four scores was compared using the McNemar test. Statistical analyses were performed using Medcalc software, version 20.014 (MedCalc Software Ltd, Ostend Belgium).

## Results

About 286 adolescents [mean age 14.3 years ± 2.5; 154 (53.6%) males] underwent liver biopsy for NAFLD between 2014 and 2019 at the Hepatology Unit of Bambino Gesù Children's Hospital. The characteristics of the population are shown in Table 1.

**Table 1:** Anthropometric, laboratory characteristics, and scores values of population.

**Variables**	**Mean (SD) or Median (25th−75th centile)**
Age, years	14.1 (1.8)
Sex (M/F)%	132/154(46.2/53.8)
BMI, kg/sqm	29.1 (5.9)
WC, cm (IQR)	91.4 (86–98)
Uric acid, mg/dl	5.8 (1.4)
Albumin, g/dl	3.9 (0.5)
Platelets, (IQR)	245 (179–302)
ALT, UI/L (IQR)	51 (21–65)
AST, UI/L (IQR)	34 (21–40)
GGT, UI/L (IQR)	24.9 (13–30)
Total-chol, mg/dL (IQR)	151 (130–180)
HDL, mg/dL(IQR)	45.4 (37–50)
Triglycerides, mg/dL (IQR)	134 (73–150)
Glucose, mg/dL (IQR)	83.7 (77–97)
Insulin, μUI/ML (IQR)	18.6 (10–29)
HOMA-IR	3.8 (2.6)
DBP, mmHg (IQR)	66 (54–78)
SBP, mmHg (IQR)	116 (108–124)
**Fibrosis score**
APRI (IQR)	1.22 (0.81–2.14)
FIB-4 (IQR)	0.35 (0.21–0.47)
Hepamet (IQR)	0.42 (0.12–0. 67)
NFS (IQR)	0.60 (−1.5 to −0.71)
PNFI (IQR)	5.88 (2–9.5)

Our population showed a fibrosis score ≥ 1 in 173 (60.4%) adolescents. In particular: 140 (49.3%) patients had *F* = 1, 31 (10.8%) had *F* = 2 and 2 (0.66%) had *F* = 3 (Table 2). In addition, HOMA-IR was pathological in 108 (37.6%), while 22 others (7.6%) were diagnosed with impaired fasting glucose (IFG) and in 3 (1%) with T2DM.

**Table 2:** Histological characteristics of the population.

**Steatosis**		
0	24	8.4%
1	70	24.5%
2	113	39.5%
3	79	27.6%
**Portal inflammation**
0	73	25.5%
1	185	64.7%
2	28	9.8%
**Lobular inflammation**
0	81	28.3%
1	140	49%
2	65	22.7%
**Ballooning**
0	88	30.8%
1	125	43.7%
2	73	25.5%
**Fibrosis**
0	105	36.7%
1	140	49%
2	31	10.8%
3	2	0.6%
**NAS**
0	14	4.9%
1	23	8%
2	46	16.1%
3	50	17.5%
4	19	6.6%
5	81	28.3%
6	45	15.7%
7	8	2.8%


Median values of APRI was 1.2 (0.8–2.1) expressing an increased probability of significant fibrosis (APRI > 1). For FIB-4, the median (0.8, 0.2–1.1) was below the cut-off value of fibrosis (FIB-4 > 1.45), as well as for NFS (0.6, −1.5 to −0.71) (cut-off > 0.675), Hepamet (0.42, 0.12–0.67) (cut-off > 0.47), and PNFI (5.88, 2–9.5) (cut-off > 9) (Table 1).

By assessing the population based on the degree of fibrosis, patients with F > 2 had a significantly lower value of platelets, even if still in a normal range. In addition, they showed significantly worse lipid (cholesterol and triglycerides) and glycoinsulinemic (insulin and HOMA-IR) profiles, compared to the other two groups. On the other hand, patients with different severity of fibrosis had similar anthropometric parameters (age, gender, BMI, and waist circumference). Patients without fibrosis showed a median value of APRI, FIB-4, Hepamet, NFS, and PNFI of 0.6 (0.4–1.1), 1.1 (0.9–1.2), 0.19 (0.1–0.5),0.3 (−1.4 to 0.5), and 4.6 (1.1–7), respectively. A statistically significant difference in median fibrosis score values was found between patients without fibrosis and those with mild (F1) or moderate-severe fibrosis (>F2) for all scores but FIB-4 which was not able to detect differences between the 3 groups of patients divided according to fibrosis severity (Table 3).

**Table 3:** Characteristics of the population according to the degree of fibrosis.

	**F0 (N 105)**	**F1 (N 140)**	**F > 2 (N 33)**	***P***
Age, years	14.1 (1.6)	14.1 (1.7)	14.2 (1.6)	0.67
Sex, (F/M),%	47.6/52.4	50/50	36.3/63.7	0.77
BMI, kg/sqm	27.8 (5.1)	28.9 (5.6)	29.3 (6.1)	0.75
WC, cm (IQR)	89 (78–93)	90 (85–98)	92 (86–99)	0.82
Uric acid, mg/dl	5.4 (1.2)	5.8 (1.4)	6.2 (1.5)	0.26
Albumin, g/dl	4.1 (0.6)	3.9 (0.5)	3.8 (0.5)	0.79
Platelets, 10^9^ (IQR)	279 (190–344)	277 (179–302)	198 (178–302)	0.01
ALT, UI/L (IQR)	42 (21–58)	50 (20–65)	55 (22–68)	0.05
AST, UI/L (IQR)	34 (19–41)	34 (21–40)	36 (21–42)	0.08
GGT, UI/L (IQR)	20 (11–32)	18 (13–29)	26 (14–32)	0.18
Total-chol, mg/dl (IQR)	161 (75–178)	157 (78–183)	197 (77–213)	0.04
HDL, mg/dl (IQR)	40 (35–48)	41 (35–50)	38 (36–52)	0.10
Triglycerides, mg/dl (IQR)	110 (68–164)	112 (73–141)	136 (73–155)	0.02
Glucose, mg/dl (IQR)	81 (70–87)	83 (76–89)	91 (77–99)	0.04
Insulin, μUI/ML (IQR)	17 (10–22)	18 (11–23)	22 (10–28)	0.03
HOMA-IR (IQR)	3.5 (2.2–4.1)	3.8 (2–4.8)	5.5 (2.1–6.5)	0.04
DBP, mmHg (IQR)	65 (55–75)	65 (58–72)	66 (59–74)	0.27
SBP, mmHg (IQR)	114 (105–120)	115.7 (108–123)	116 (108–124)	0.45
**Fibrosis score**
APRI (IQR)	0.6 (0.4–1.1)	1.2 (0.8–1.9)	1.5 (0.9–2.1)	0.02
FIB-4 (IQR)	1.1 (0.9–1.2)	1.4 (0.8–1.6)	1.5 (0.5–2.1)	0.07
Hepamet (IQR)	0.19 (0.1–0.5)	0.41 (0.1–0.8)	0.68 (0.2–0.8)	0.02
NFS (IQR)	0.3 (−1.4 to 0.5)	0.3 (−1.4 to 0.7)	0.7 (−1 to 0.8)	0.04
PNFI	4.6 (1.1–7)	6.5 (3–9)	7.5 (4–9.5)	0.02

### Correlations Between NAFLD Scores

In correlation analysis of the fibrosis scores with the main anthropometric and laboratory parameters resulted that Hepamet and NFS were correlated with BMI, while no correlations were found between APRI and FIB-4 and BMI. All four scores correlate with ALT serum activity (<0.05).

Hepamet correlates with HOMA-IR (*r* = 0.27, *p* = 0.001) and with triglycerides (*r* = 0.26, *p* = 0.008), as well as fibrosis (HOMA-IR *r* = 0.18, *p* = 0.001; Triglycerides *r* = 0.17, *p* = 0.002), while NFS only with triglycerides (*r* = 0.12, *p* = 0.002). Platelets correlate with APRI and FIB-4 (*p* < 0.05; Table 4).

**Table 4:** Correlations between NAFLD scores, fibrosis and anthropometric and laboratory characteristics.

	**APRI**	**FIB-4**	**HEPAMET**	**NFS**	**FIBROSIS**
SEX	r	−0.07	−0.07	0.03	0.02	0.081
	p	0.18	0.23	0.95	0.72	0.16
AGE IN YEARS	r	−0.13	−0.07	−0.11	−0.17	0.34
	p	0.01	0.23	0.045	0.003	<0.0001
BMI	r	−0.15	−0.13	0.18 am	0.20 a.m.	0.21
	p	0.11 am	0.12 am	04.04 am	0.03 am	0.03
URIC ACID	r	−0.04	−0.03	−0.04	−0.01	0.13
	p	0.49	0.60	0.41	0.80	0.05
ALBUMIN	r	0.012	−0.04	0.03	0.011	−0.06
	p	0.99	0.94	0.57	0.85	0.30
ALT	r	0.14	0.02	0.17	0.20	−0.06
	p	0.01	0.70	0.002	0.06	0.24
AST	r	0.18	0.07	0.15	0.16	−0.14
	p	0.001	0.22	0.08	0.07	0.01
GGT	r	0.14	0.06	0.12	0.14	−0.09
	p	0.01	0.31	0.03	0.01	0.13
GLUCOSE	r	0.004	0.02	0.16 am	−0.06	0.04
	p	0.94	0.69	0.09 am	0.28	0.46
HDL-CHOL	r	0.07	0.06	−0.03	−0,07	−0.15
	p	0.23	0.26	0.54	0.20	0.007
HOMA-IR	r	−0.04	−0.03	0.27 am	0.09	0.18
	p	0.47	0.61	0.001 am	0.12	0.001
TRIGLYCERIDES	r	0.10	0.09	0.26 am	0.11	0.17
	p	0.90	0.60	0.008	0.002	0.002
INSULIN	r	−0.04	−0.04	0.17 am	−0.02	0.27
	p	0.49	0.48	04.04 am	0.73	0.0001
DBP	r	0.07	0.06	0.11 am	0.12 am	0.1
	p	0.22	0.27	0.21 am	0.10 am	0,09
SBP	r	−0.07	−0.07	0.09	0.10 am	0.11
	p	0.21	0.22	0.10	0.11 am	0.05
PLATELETS	r	−0.22	−0.23	−0.001	−0.04	−0.004
	p	0.0002	0.0001	0.98	0.47	0.94
FIBROSIS	r	−0.03 0.58	−0.06	0.74		0.13
	p		0.27	<0.0001		0.02
NAS	r	0.02	0.01 0.86	0.25	0.34	0.30
	p	0.63		<0.0001	<0.0001	<0.0001

### Comparison of Non-Invasive Fibrosis Scores

**Figure 1:**
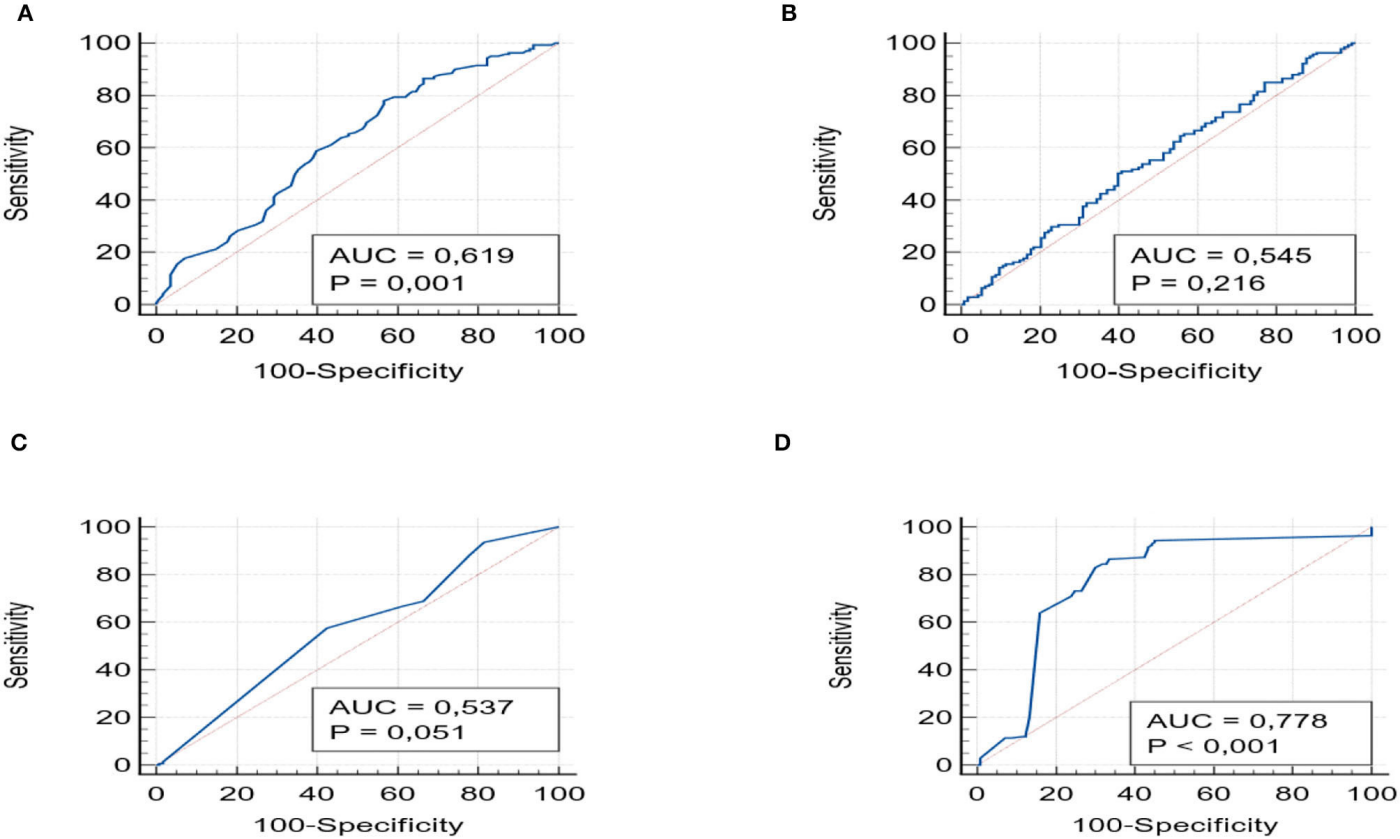
shows the ROC curves with the AUROCs for APRI **(A)**, FIB-4 **(B)** NSF **(C)**, and Hepamet **(D)**, with the values of *p*.

The values of the area under the curve (AUROC) were obtained for all scores: APRI, FIB-4 index, NFS, and Hepamet which were then compared with the degrees of fibrosis. APRI and Hepamet were found to be good predictors of fibrosis. APRI (AUROC 0.61, 95% CI 0.556–0.679) and Hepamet (AUROC 0.77, 95% CI 0.722–0.828) (*p* < 0.001) had significant accuracy to distinguish subjects with fibrosis. On the other hand, the NFS (AUROC 0.53; 95% CI 0.510–0.635, *p*=0.05) and FIB-4 (AUROC 0.54; 95% CI 0.482–0.608, *p* = 0.12) score had poor diagnostic accuracy for fibrosis (Figures 1A–D).

For each of the four scores, we also evaluated the PPV and NPV values for detecting any fibrosis (F > 1). These were similar among different scores. APRI had a PPV of 62.77% (95% CI 57.96–67.35) and an NPV of 52.01% (95% CI 46.54–57.43) and Hepamet a PPV of 63.24% (95% CI 59.95–66.41) and an NPV of 61.29% (52.9–69.01). FIB-4 and NFS showed the same PPV (62%; 95% CI 57–67) and NPV (52%; 95% CI 46.5–57.4) values.

**Figure 2:**
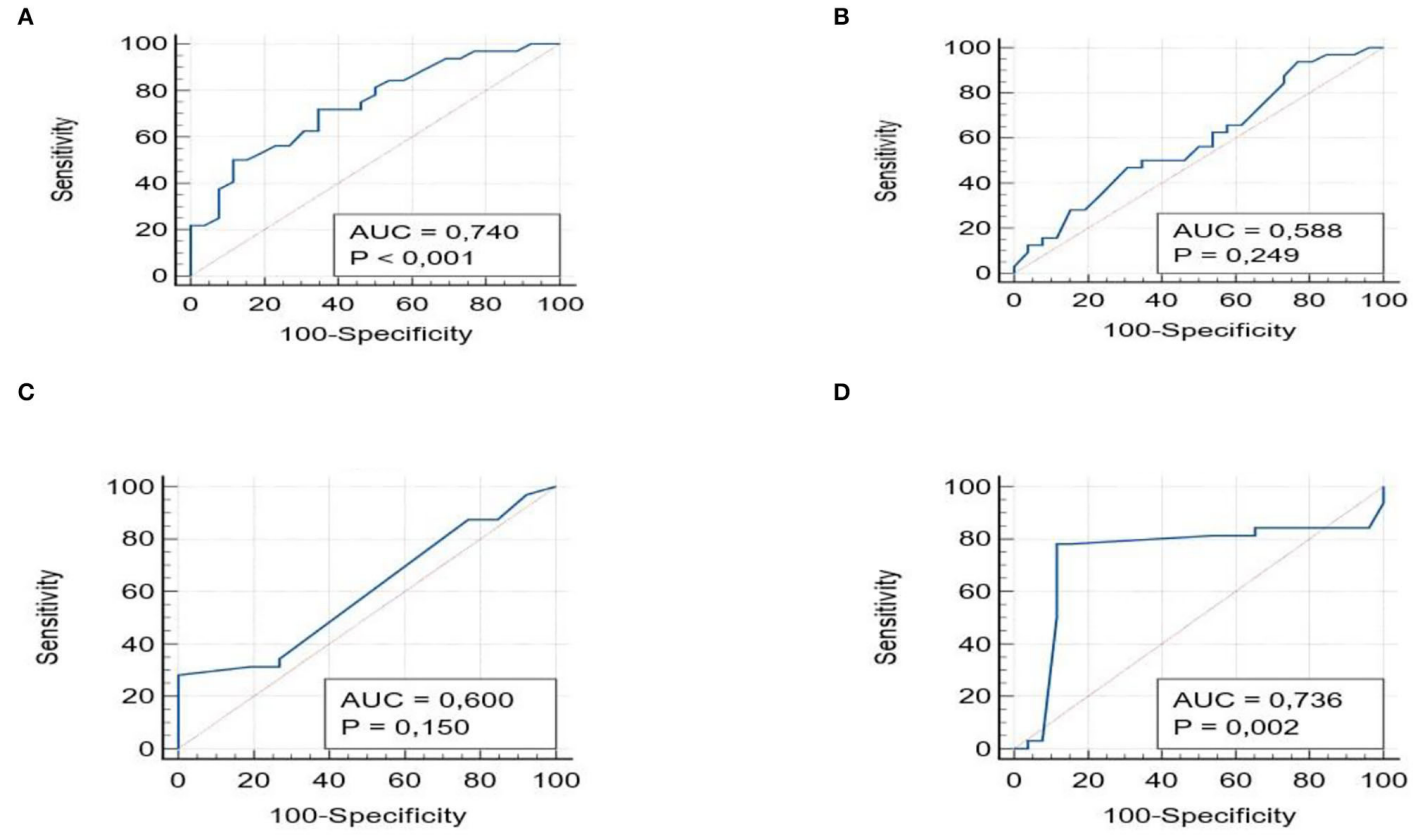
ROC curves with the AUROCs for APRI **(A)**, FIB-4 **(B)**, NSF **(C)**, and Hepamet **(D)** for Fibrosis ≥2.

Moreover, we conducted ROC analysis for detecting patients with moderate to severe fibrosis (F > 2). The AUROCs for APRI (0.74, 95% CI 0.60–0.84) and Hepamet (0.73, 95% 0.62–0.84) were always significant (*p* < 0.001), with PPV of 86% and 88.8%, NPV of 78.1 and 76.6%, respectively; while FIB-4 (0.58, 95% CI 0.45–0.71), and NFS (0.60, 95% CI 0.46–0.72) (*p* > 0.05) showed non-significant AUROC (Figures 2A–D).

**Figure 3:**
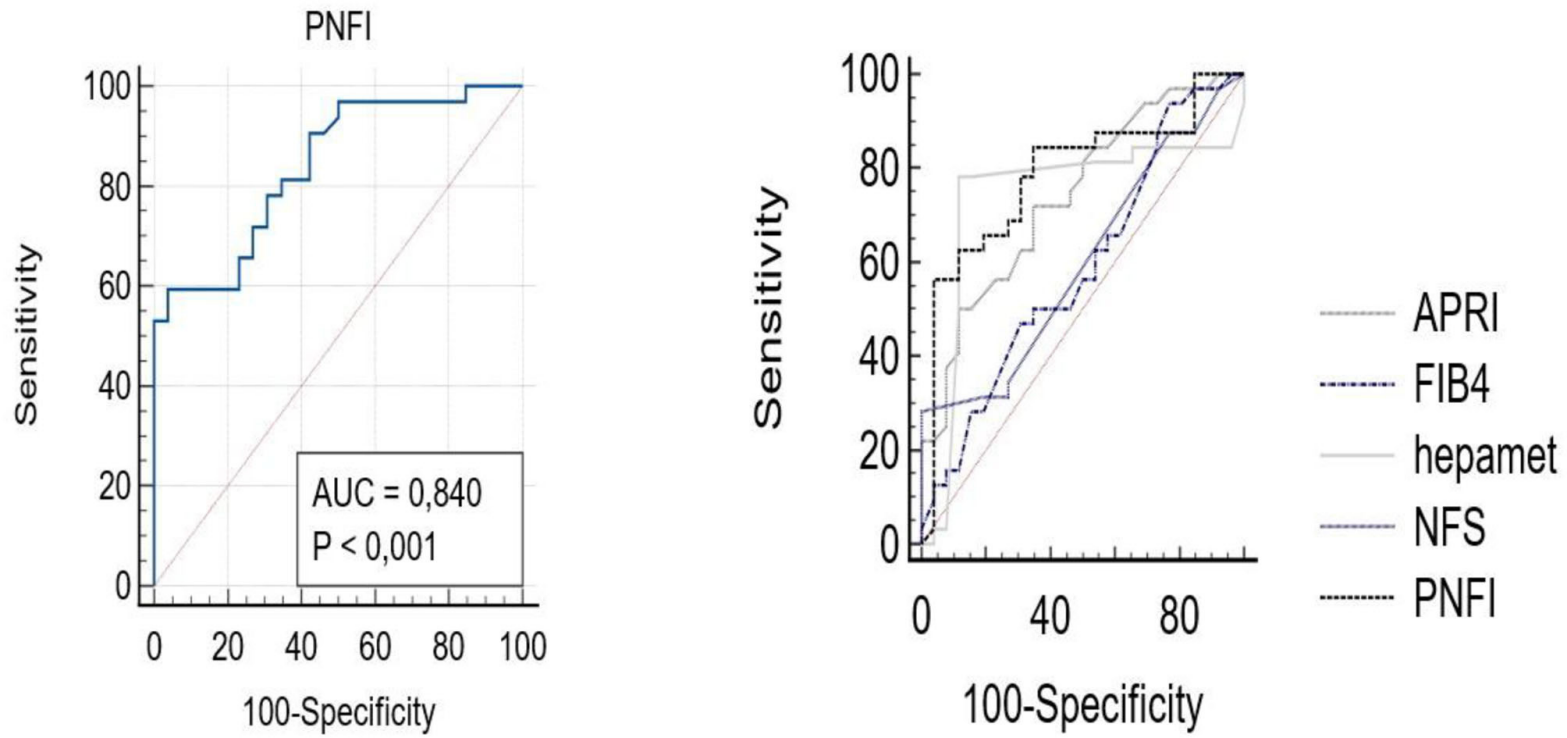
Comparison between the ROC curves for all scores and PNFI.

Finally, we performed the ROC curves for the PNFI, a score developed for the pediatric age. The AUROC (F > 1)was 0.81 (95% CI 71.3–83.2), with PPV 90.25% (95% CI 88.7–99.1) and NPV 75.4% (95% CI 72.1–82.4). For the F > 2 group, the AUROC was 0.84 (95% CI 0.75–0.92) with PPV 97.5% (89–99.3) and NPV 72.6 (95% 67–81). Compared with the other scores, it remained the most significant for the pediatric age, especially compared to FIB-4 and NFS (*p* < 0.05; Figure 3).

## Discussion

The prevalence of NAFLD has increased worldwide, affecting especially children in the Western world. Since to date for the diagnosis of NASH, biopsy remains the gold standard, we wanted to investigate whether the NAFLD scores used in the adult population can be used as non-invasive fibrosis tests (7, 22).

Our study is, therefore, the first pediatric study to cover a large population of adolescents undergoing liver biopsy for NAFLD with F > 1 finding in 63.2% of histological examinations; this allowed us to test the four non-invasive scores of NAFLD.

We found that among the four different non-invasive fibrosis scores only APRI and Hepamet were shown to have significant predictive value for diagnosing children with fibrosis as a complication of NAFLD. The NFS and FIB-4 indices showed poor performance as diagnostic markers for clinically significant fibrosis.

These data confirm what is known in the literature that APRI can be used in the pediatric population, probably because it does not consider age and BMI unlike NFS and FIB-4 (11). Probably, as we have shown with the study of correlations, the trend of NFS was associated with BMI, while FIB-4 correlates with platelets alone, which in children with NAFLD are hardly reduced.

In addition, in our population, APRI and Hepamet have exhibited promising results for predicting the presence of fibrosis (F1–F4) with AUROC = 0.61 and = 0.77, respectively. This is in accordance with previous studies by Rigamonti et al. and Mansoor et al. who have shown similar results, in which, APRI was significantly different among patients with mild fibrosis and moderate/severe fibrosis (APRI, *p* = 0.032) (11, 21, 22).

An important result was obtained with the use of Hepamet which had significant accuracy to distinguish pediatric patients with fibrosis (AUROC 0.778, 95% CI 0.722–0.828) (*p* < 0.001). A recent study showed, in a Mexican adult population, that the Hepamet score at a high NPV (90.1%, 95% CI 85–93. 9), was useful to exclude advanced fibrosis, while PPV was limited at 36.7% (95% CI 19. 9–56. 1). In our study, Hepamet showed a PPV of 63.24% (95% CI 59.95–66.41) and a NPV of 61.29% (52.9–69.01) for values of fibrosis > 1, significant, though not optimal, predictive values (23, 24).

Furthermore, we tried to perform the curves for the small population with advanced fibrosis (F≥2). This is because grade 1 fibrosis is the most frequent but not clinically significant. In this population, AUROC values always show the significance for APRI (0.74, 95% CI 0.60–0.84) and Hepamet (0.73, 95% 0.62–0.84), but with a higher AUROC value for APRI compared to Hepamet, with higher NPV (78.1 and 76.6%) than the F > 1 population.

Moreover, comparing these AUROCs with that of the PNFI, our data shows that the PNFI remains the best non-invasive score in pediatric age.

We believe that the APRI score may be useful in pediatric age to evaluate the possible presence of F > 1, but Hepamet may also play a role as the pediatric population affected by NAFLD is in most cases related to visceral obesity (70% of cases) and insulin resistance, variables used in this score (Table 1).

However, we believe that the Hepamet has limits in the evaluated variables that include age, diabetes, and HOMA-IR, unlike the APRI, which weighs heavily in the final score (13, 23, 24). In our population the presence of high HOMA-IR is significant, but the mean age is very low, as well as the incidence of diabetes, factors that will certainly have influenced the final score.

In fact, both in the general population and in the stratification according to the degree of fibrosis, our patients have significantly high values of BMI and HOMA-IR, which we know to be among the first “shots” at the base of the pathogenesis of pediatric NAFLD, correlating with inflammation and oxidative stress.

Unfortunately, among the other scores, we evaluated in our study, the FIB-4 index and the NFS were shown to have poor predictability for liver fibrosis in NAFLD children. Therefore, while these scores show very promising results in adult studies (FIB-4, AUROC: 0. 86; NFS, AUROC = 0. 81; AST/ALT ratio, AUROC = 0. 83), the same cannot be affirmed for adolescents with NAFLD, based on data from our study (*p* > 0.05).

One of the limitations of this study is the degree of fibrosis, since the population with F > 2 represented only 10.8%, while all previous papers on Hepamet, FIB-4, and NFS scores have focused on the stage of fibrosis ≥ F3, which in the pediatric population is rare. In addition, our study sample consisted of children seen in a tertiary care center, this potentially provides a distorted representation of the true prevalence of each stage of liver fibrosis in NAFLD children, as patients are more likely to present with advanced liver disease. One advantage though is that our study has a very large sample of adolescents undergoing a liver biopsy, and the histology has been evaluated by the same pathologist in recent years. This data is important since the determination of an F1 is sometimes a dependent operator.

In conclusion, our study showed that Hepamet and APRI scores perform better than NFS and FIB-4 for identifying significant fibrosis in patients with NAFLD but do not have PPVs so high to be considered diagnostic tool. Therefore, they cannot be considered fable for certain diagnoses of fibrosis or its progression in children and cannot replace liver biopsy as the gold standard of diagnosis. It is, therefore, necessary to continue to research and develop new markers of exclusive fibrosis, which could significantly reduce the health expenditure associated with the early diagnosis of fibrosis in NAFLD but also significantly reduce the use of liver biopsy which is not entirely free of complications in children. Further studies are needed to approve the use of APRI and Hepamet in the pediatric population as well.

## Data Availability Statement

The raw data supporting the conclusions of this article will be made available by the authors, without undue reservation.

## Ethics Statement

The studies involving human participants were reviewed and approved by Bambino Gesù Children's Hospital Local Ethics Committee (Protocol Number: 539/RA, 01/07/2013). Written informed consent to participate in this study was provided by the participants' legal guardian/next of kin.

## Author Contributions

AM and GM conceptualized and designed the study, contributed to the discussion, and critically revised the manuscript. AA, AM, and GM analyzed data, interpreted results, and drafted the manuscript. AM, LD, PF, and SV enrolled patients, collected data, and revised the manuscript. All authors approved the final manuscript as submitted and agree to be accountable for all aspects of the work.

## Conflict of Interest

The authors declare that the research was conducted in the absence of any commercial or financial relationships that could be construed as a potential conflict of interest.

## Publisher's Note

All claims expressed in this article are solely those of the authors and do not necessarily represent those of their affiliated organizations, or those of the publisher, the editors and the reviewers. Any product that may be evaluated in this article, or claim that may be made by its manufacturer, is not guaranteed or endorsed by the publisher.
